# Carnitine

**DOI:** 10.1016/j.advnut.2024.100336

**Published:** 2024-11-06

**Authors:** Xi Lin, Sean H Adams, Jerry Vockley, Jack Odle

**Affiliations:** 1 Department of Animal Science, North Carolina State University, Raleigh, NC, United States; 2 Department of Surgery, University of California, Davis, School of Medicine, Sacramento, CA, United States; 3 Department of Human Genetics, University of Pittsburgh, Pittsburgh, PA, United States

Carnitine [C_7_H_15_NO_3_; (S)-3-hydroxy-4-(trimethylammonio)butanoate] is a water-soluble nutrient with a fixed quaternary ammonium. It was initially discovered as a growth factor for mealworms (genus *Tenebrio*) and later named vitamin Bt. A primary function of carnitine is the transport of long-chain fatty acids into mitochondria for β-oxidation in support of ATP production as a cellular energy currency. Transport is completed through the mitochondrial carnitine–acylcarnitine cycle involving 3 enzymes: carnitine palmitoyltransferase I (CPT I), carnitine-acyl-carnitine translocase, and carnitine palmitoyltransferase II (CPT II). In addition, carnitine protects the cell from accumulation of coenzyme A (CoA) derivatives (i.e. acyl-CoAs) via the generation of acylcarnitines and maintains a dynamic stabilization of acetyl-CoA and CoA concentrations through the capability of exchanging an acetyl group. Carnitine synthesis is adequate in healthy adults but is inadequate in neonates and individuals with certain inherited disorders of metabolism. In the latter, metabolic intermediates such as CoA-esterified metabolites accumulate, with conversion to carnitine esters and subsequent reduction of free carnitine pools.

## Deficiencies

Healthy individuals (including adults and children) with no metabolic disorders, even vegetarians, are rarely deficient in carnitine. However, people with inborn errors of metabolism and infants, especially preterm infants, can have low levels of carnitine because of inadequate de novo biosynthesis to account for urinary losses [1]. Poor carnitine status impairs mitochondrial β-oxidation, which can promote hepatic steatosis and hypoketotic hypoglycemia. The latter results from blunted ketogenesis concurrent with depression of gluconeogenesis (a pathway that utilizes ATP from fat oxidation and has key enzymes positively regulated by β-oxidation-derived NADH and acetyl-CoA). Carnitine deficiency [2] is defined as either primary or secondary. Primary deficiency is due to impaired cellular carnitine transport associated with mutations in the genes of organic cation transporters for carnitine encoded by *SLC22A4* and *SLC22A5*, especially given their prevalence, or by mutation of *SLC22A5*. Primary carnitine deficiency presents symptoms of cardiomyopathy, skeletal myopathy, and hypoglycemia with extremely low carnitine concentrations in blood and tissues. Secondary carnitine deficiency results from multiple disorders related to low carnitine intake or excessive carnitine loss due to chronic renal excretion or failure with dialysis. Secondary deficiency presents with symptoms of muscle weakness, tiredness, low blood sugar, and heart issues.

## Dietary Recommendations

Healthy individuals can synthesize all of the carnitine needed, and no recommended daily intake has been established. Carnitine can be classified as a conditionally essential nutrient, meaning that, although most individuals can synthesize enough to meet their metabolic needs, under some circumstances, endogenous synthesis is inadequate. Developing neonates, patients with selected inherited metabolic disorders, and dialysis patients, as well as individuals with a strict vegan diet, may require dietary or supplemental carnitine. Supplements are available for oral carnitine and acetyl-carnitine with no prescription, and the dose ranges from 0.5 to 2 g/d.

## Food Sources

The main sources of carnitine are meat, milk, and dairy products; carnitine is very high in red meat (≤1500 μg/g ground beef or steak) and intermediate in dairy products (≤35 μg/g whole milk or cheese) and chicken breast (≤36 μg/g). A few plant foods contain very limited amounts of carnitine such as avocado (4.3 μg/g) and asparagus (2 μg/g); otherwise, vegetables, fruits, and most other plants are devoid of carnitine. Synthetic L-carnitine, acetyl-carnitine, and propionyl-L-carnitine are available in dietary supplement pills or powders, with a range of 0.5–1 g/d considered safe. The bioavailability of dietary carnitine averages 70%.

## Clinical Uses

Carnitine, as a clinical supplement, has been approved for the treatment of primary/secondary carnitine deficiency caused by mutation in the genes that code for carnitine transporters, inborn errors of metabolism that can lead to carnitine loss such as organic acidemias and fatty acid oxidation disorders, and in patients with end-stage renal disease undergoing hemodialysis. Supplementation of symptomatic patients with carnitine (100–300 mg/[kg/d]) resolves the systemic manifestations of carnitine transporter defect and prevents additional episodes of metabolic decompensation. Its use in secondary carnitine deficiency seems logical, but formal data are lacking. Doses are usually lower than for carnitine transporter deficiency (50–100 mg/[kg/d]). Carnitine is frequently prescribed as a component of a variable cocktail of medications used in patients with inborn errors of the mitochondrial respiratory chain, but again there are no formal studies documenting efficacy. Two other carnitine derivatives have received some use in clinical and research settings. Propionylcarnitine has been proposed as a treatment for cardiac dysfunction, especially in the context of ischemia, chronic fatigue, and some neurologic conditions; however, results in all cases have been inconclusive or conflicting, and thus the compound should continue to be considered only as a research agent. Similarly, acetyl-carnitine has been proposed to act as an antioxidant and has been used in mitochondrial respiratory chain deficiencies, neurodegenerative disorders, and to slow aging. Again, no conclusive evidence supports efficacy.

## Toxicity

Carnitine is well tolerated because excesses are readily excreted into the urine. However, taking ≥3 g/d may cause nausea, vomiting, abdominal cramps, diarrhea, and body odor smelling like fish. It also can cause skin rash, muscle weakness, or seizures in people with epilepsy. Overconsumption of carnitine can lead to greater production of trimethylamine via gut microbiota and trimethylamine N-oxide (TMAO) after being transformed by flavin-containing monooxygenase in the liver [3]. High TMAO has been associated with adverse cardiovascular events such as heart failure, myocardial infarction, and stroke. However, these associations appear to be context-specific, because consumption of fish can also significantly increase TMAO despite reductions in cardiometabolic diseases with higher intakes of fish.

## Recent Research

Given carnitine’s central role in bioenergetics, a plethora of studies have examined its biology across a wide array of applications. Some examples include

• Fat oxidation effects. Defects in β-oxidation and mitochondrial oxidative phosphorylation impacting global neuromuscular function, response to pathogens, tumor progression, and anesthesia [4]
• Neurologic effects. Neuroprotection via impacts on neurotransmitters in the central nervous system, improved myelination, traumatic brain injury, Alzheimer’s disease, depression [5]
• Muscle effects. Exercise recovery, chronic fatigue, cardiac hypoxic ischemic injury, cardiomyopathy [6]
• Protein binding. Long-chain acylcarnitines (LCACs) bind to myoglobin and possibly other lipid-binding proteins in myocytes [7]

Use of plasma carnitine and LCACs as clinical mitochondria biomarkers in patients have been explored extensively using metabolomics techniques [7]. Acylcarnitine profiles have been used to assess disease states (e.g., inherited disorders of metabolism, diabetes, sepsis, cancer, heart failure) and to prognosticate mortality. Decreases in LCAC with 12–14 carbons could be promising diagnostic biomarkers in the pathophysiology of Parkinson’s disease. Accumulation of LCAC with ≥14 carbons could be correlated to the stage and severity of cardiovascular disease including heart failure, coronary artery disease, and cardiac arrhythmias, and LCAC concentrations are high in sites of cardiac ischemia injury. The excessive accumulation of LCAC is related to lipotoxicities including mitochondrial membrane hyperpolarization, stimulation of reactive oxygen species production in cardiac mitochondria, insulin resistance, and cell stress responses as reflected by interleukin-6 (IL-6) production and caspase-3 activation. LCACs are detectable in normal human lung lavage fluid and accumulate at the air-fluid interface in lungs of individuals with long-chain acyl-CoA dehydrogenase deficiency. Thus, excess LCAC may represent a risk factor for lung injury in humans with dysfunctional fatty acid oxidation, although this phenomenon has not been generally observed in these patients.

Carnitine acts in vitro as an antioxidant [8] to prevent oxidative damage, reduce inflammation [9], and inhibit apoptosis under oxidative stress. Carnitine as a potential antioxidant improves oocyte quality and embryonic development. Carnitine consumption may help critically ill patients with pemphigus vulgaris and benefits the management of patients with sepsis/septic shock. These findings are believed to be associated with an increase in total antioxidant capacity, alanine transaminase, and aspartate transaminase and decrease in C-reactive protein, interleukin-6, tumor necrosis factor-α, and malondialdehyde in the serum

## Author contributions

LX wrote the initial draft. SHA, JV and JO added content and edits to the manuscript. All approved the final version.

## Conflicts of interest

SHA is founder and principal of XenoMed, LLC (dba XenoMet), which is focused on research and discovery in the area of microbial metabolism. XenoMed had no part in the research design, funding, results, or writing of the manuscript. All other authors report no conflicts of interest.

## Funding

The authors reported no funding received for this study.
